# Preventive effect of intermittent cerebrospinal fluid drainage for secondary chronic hydrocephalus after aneurysmal subarachnoid hemorrhage

**DOI:** 10.1186/s12987-023-00486-5

**Published:** 2023-12-06

**Authors:** Tomoyasu Yamanaka, Yusuke Nishikawa, Takashi Iwata, Teishiki Shibata, Mitsuru Uchida, Yuki Hayashi, Hiroyuki Katano, Motoki Tanikawa, Shigeki Yamada, Mitsuhito Mase

**Affiliations:** 1 Department of Neurosurgery, Graduate School of Medical Science, Nagoya City University, Kawasumi 1, Mizuho-cho, Mizuho-ku, Nagoya City, Aichi 467-8601 Japan; 2 Department of Neurosurgery, Nagoya City East Medical Center, Aichi, Japan; 3 Department of Neurosurgery, Nagoya City West Medical Center, Aichi, Japan; 4 Interfaculty Initiative in Information Studies, Institute of Industrial Science, The University of Tokyo, Tokyo, Japan

**Keywords:** CSF drainage, Subarachnoid Hemorrhage, Coil embolization, Chronic hydrocephalus, Acute hydrocephalus

## Abstract

**Background:**

The efficacy of intermittent cerebrospinal fluid (CSF) drainage compared with that of continuous CSF drainage in patients with subarachnoid hemorrhage (SAH) remains undetermined to date. Therefore, we investigated whether intermittent CSF drainage is effective in reducing secondary chronic hydrocephalus (sCH) after aneurysmal SAH.

**Methods:**

Overall, 204 patients (69 men and 135 women) treated for aneurysmal SAH between 2007 and 2022 were included in this study. Following SAH onset, 136 patients were managed with continuous CSF drainage, whereas 68 were managed with intermittent CSF drainage. Logistic regression analyses were used to calculate the age-adjusted and multivariate odds ratios for the development of sCH. The Cox proportional hazards regression model were used to compare the effects of intermittent and continuous CSF drainage on sCH development.

**Results:**

Overall, 96 patients developed sCH among the 204 patients with SAH. In total, 74 (54.4%) of the 136 patients managed with continuous CSF drainage developed sCH, whereas 22 (32.4%) of the 68 patients managed with intermittent CSF drainage developed sCH. This demonstrated that the rate of sCH development was significantly lower among patients managed with intermittent CSF drainage. Compared with continuous CSF drainage, intermittent CSF drainage exhibited a multivariate odds ratio (95% confidential interval) of 0.25 (0.11–0.57) for sCH development. Intermittent CSF drainage was more effective (0.20, 0.04–0.95) in patients with severe-grade SAH than in those with mild-grade SAH (0.33, 0.12–0.95). Intermittent CSF drainage was ineffective in patients with acute hydrocephalus (8.37, 0.56–125.2), but it was effective in patients without acute hydrocephalus (0.11, 0.04–0.31).

**Conclusions:**

Compared with continuous CSF drainage, intermittent drainage is more effective in reducing sCH after aneurysmal SAH. Although intermittent drainage was ineffective in cases of co-occurrence of acute hydrocephalus, it was effective in reducing sCH development regardless of the severity of initial symptoms at SAH onset.

## Background

Subarachnoid hemorrhage (SAH) is recognized as one of the most lethal disorders, and SAH survivors often have a low quality of life. The poor prognosis of this condition is mainly attributed to various comorbidities, including cerebral infarction due to cerebral vasospasm and secondary chronic hydrocephalus (sCH) after SAH. Notably, regarding the prevalence of sCH, < 50% of the patients with aneurysmal SAH have been reported to have sCH [1–12]. At the onset of SAH, severe initial symptoms, a large amount of diffuse subarachnoid hematoma, and the presence of concomitant acute hydrocephalus have been linked to the development of sCH [1–14]. Furthermore, microsurgical interventions, such as widening of cisterns and fenestration of the lamina terminalis, have been reported to be associated with the development of sCH [15–17]. In 2012, the American Heart Association and American Stroke Association published guidelines for the management of aneurysmal SAH, which recommended that fenestration of the lamina terminalis should not be routinely performed to prevent sCH after aneurysmal SAH [2]. Moreover, it has been reported that compared with microsurgical clipping, coil embolization for ruptured cerebral aneurysm significantly reduces the likelihood of sCH development and provides a favorable prognosis after SAH [11]. These findings may support the hypothesis that microsurgical procedures accelerating inflammation of the arachnoid membrane may promote adhesion and fibrosis of the membrane, which is possibly related to the development of sCH.

Moreover, according to Rao et al., compared with continuous cerebrospinal fluid (CSF) drainage and gradual weaning, intermittent CSF drainage and rapid weaning of external ventricular drainage were reported to be associated with a lower number of patients with sCH requiring permanent ventriculoperitoneal shunt (VPS) implantation [18]. Further, the same group of authors recently conducted a randomized multicenter-controlled study to compare rapid and gradual weaning of external ventricular drainage [19]. They concluded that rapid weaning of external ventricular drainage is effective. However, to date, to the best of our knowledge, no study has proven that intermittent CSF drainage alone is effective. Therefore, this study aimed to determine whether intermittent CSF drainage could reduce sCH after aneurysmal SAH compared with continuous CSF drainage.

## Methods

### Patient population

**Fig. 1:**
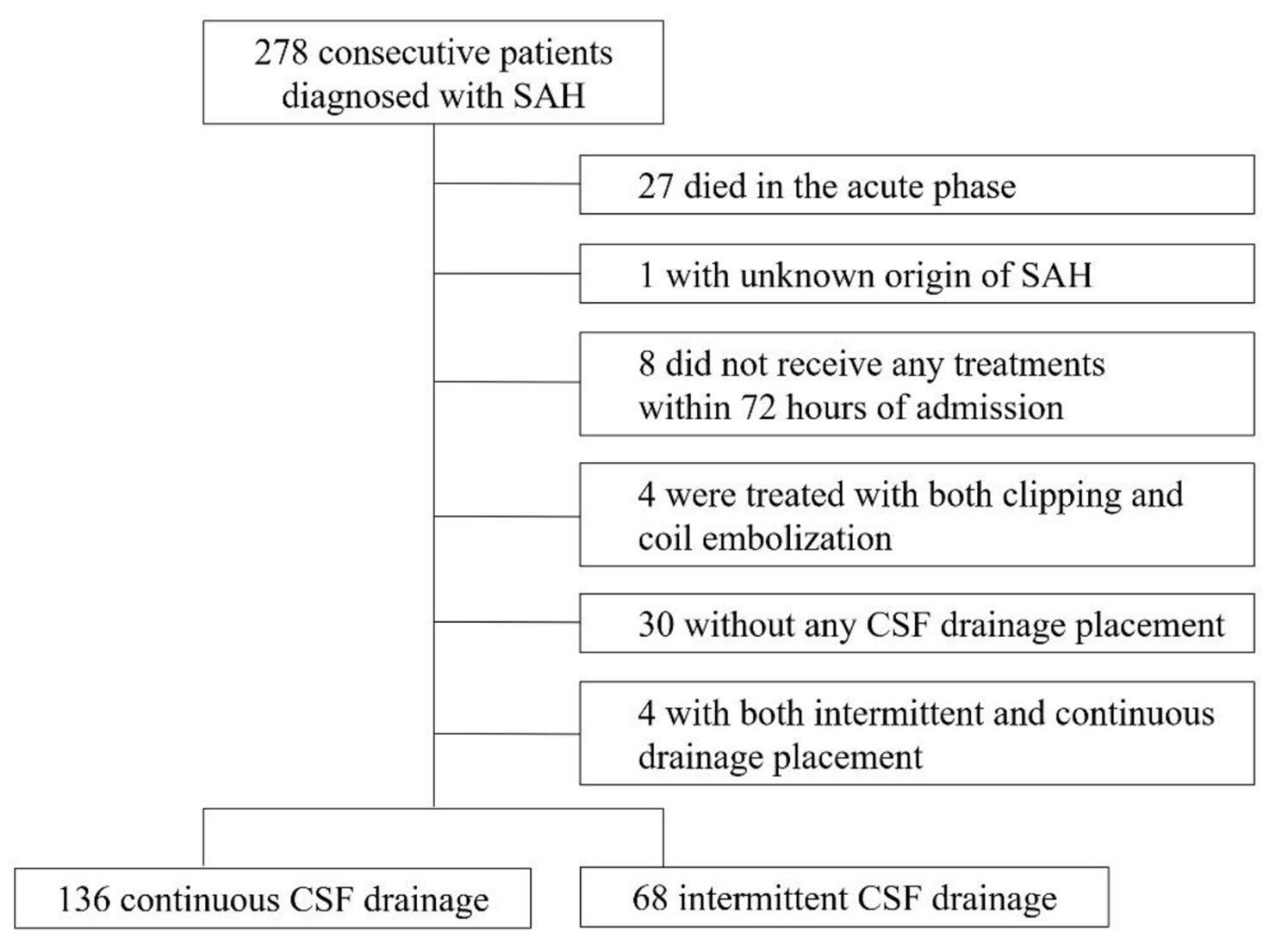
Flowchart for selection of study population

We retrospectively identified 278 consecutive patients with SAH who were admitted to Nagoya City University between February 2007 and November 2022. Of them, 27 patients died in the acute phase of SAH onset, 1 was unknown origin SAH, 8 had not received any treatments for ruptured aneurysms within 72 h of admission, 4 were treated with both microsurgical clipping and coil embolization, 30 did not undergo CSF drainage, and 4 were managed with both intermittent and continuous CSF drainage. Finally, 204 patients were enrolled in this study, including 136 patients managed with continuous CSF drainage and 68 patients managed with intermittent CSF drainage (Fig. 1).

Detailed information on CSF drainage methods (intermittent or continuous), duration of CSF drainage, and time from the onset of SAH to the development of sCH was obtained from the medical records of the patients. Other clinical data collected were as follows: age at SAH onset, sex, medication status, World Federation of Neurological Surgeons (WFNS) scale grade for initial symptoms of severity at SAH onset, Fisher rating scale grade at admission, and treatment of the ruptured aneurysm (neurosurgical clipping and/or endovascular coil embolization). The ruptured aneurysms were classified according to their location as follows: middle cerebral artery (MCA), 39; anterior communicating artery (ACoA), 57; anterior cerebral artery (ACA), 11; internal carotid-posterior communicating artery (IC-PCoA), 45; other internal carotid artery (ICA), 17; posterior cerebral artery (PCA), 3; basilar artery (BA), 6; BA–superior cerebellar artery (BA-SCA), 1; vertebral artery (VA), 18; VA–posterior inferior cerebellar artery (VA-PICA), 7. The locations of the aneurysms were divided into five categories: MCA, ACA (including ACA and ACoA), IC-PCoA, ICA, and posterior circulation (including PCA, BA, BA-SCA, VA, and VA-PICA). Concomitant acute hydrocephalus was defined as patients with ventricular enlargement on CT scan and rapidly progressive loss of consciousness requiring immediate CSF drainage via external ventricular drainage, and sCH was defined as patients with progressive ventricular enlargement on CT scan and progressive symptoms, such as impaired consciousness, requiring CSF shunt surgery if CSF removal from the ventricles or lumbar subarachnoid space was not performed at least 2 weeks after the onset of SAH. Acute hydrocephalus is often obstructive hydrocephalus, while sCH after SAH is usually classified as non-obstructive hydrocephalus.

### CSF drainage management protocol

Patients with concomitant acute hydrocephalus were immediately treated with emergency external ventricular drainage to control intracranial pressure; subsequently, treatments for ruptured cerebral aneurysms were performed. Intracranial pressure was controlled to approximately ≤ 20 cm H_2_O by adjusting the height of the drainage disk open to atmospheric pressure before treatment of ruptured cerebral aneurysms in order to reduce the risk of rebleeding. Further, after treatment, the pressure was controlled down to to approximately 10 cm H_2_O. Notably, the height of the drainage disk should be correctly adjusted when measuring the CSF drainage volume. If the intracranial pressure does not rise for several days after the SAH onset, CSF drainage method is switched from external ventricular to external lumbar drainage.

**Fig. 2:**
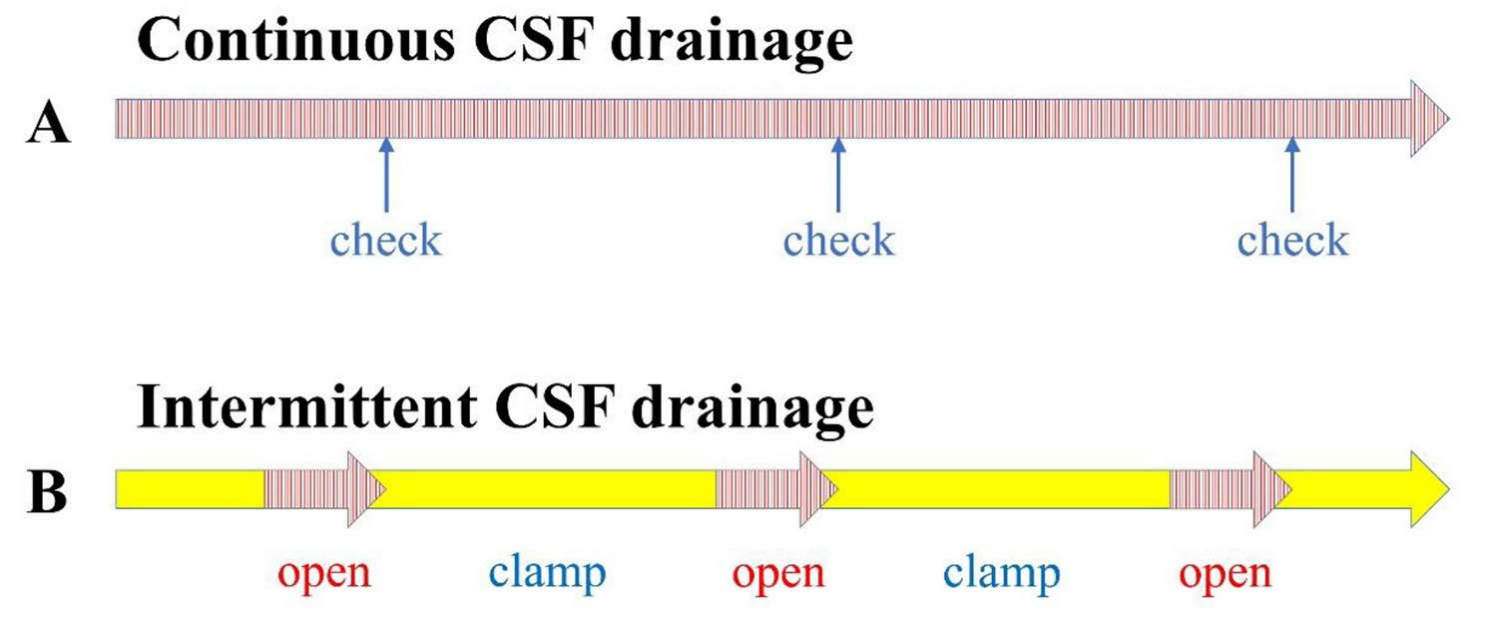
Diagrams of intermittent and continuous CSF drainage. In continuous CSF drainage (**A**), the drained CSF volume is measured every 8 h (↑). The approximate CSF drainage volume of 150 mL per day is modified by the height of the disk open to atmospheric pressure; if the CSF drainage volume within 8 h is < 40 mL, the disk is lowered by 2 cm; if the CSF drainage volume is > 60 mL, the disk is raised by 1 cm. In intermittent CSF drainage (**B**), CSF is drained three times a day, with 50 mL of CSF drained per session; disk is clamped when 50 mL of CSF is drained, and it remains clamped until the next session. If 50 mL of CSF is not drained after 4 h of opening, the height of the disk is adjusted (lowered by 2 cm), and it is clamped until the next session

In our study, the CSF drainage volume was assessed every 8 h in the continuous CSF drainage method (Fig. 2A), and disk height was adjusted, as appropriate, on the first day of external ventricular drainage to achieve the daily CSF drainage volume and switched to intermittent CSF drainage after 24 h if there was no sharp increase in intracranial pressure. Patients with SAH and remarkable concomitant intracerebral hematomas or consistently high intracranial pressure were not indicated for intermittent CSF drainage and managed with continuous CSF drainage. Otherwise, intermittent CSF drainage was performed three times per day, with pressure adjusted to achieve 50 mL of the CSF drainage volume in approximately 2 h per session (Fig. 2B). If 50 mL of the CSF had not been drained after 4 h, the pressure was reduced by 2 cm H_2_O, and the drainage was clamped until the next session. During intermittent CSF drainage, if the patients were in good condition, i.e., if they were conscious and able to move, they were allowed to leave the bed with the drain clamped and actively engage in rehabilitation interventions, such as standing and gait training. In contrast, during continuous CSF drainage, the patients remained in the bed and uderwent rehabilitation therapies to prevent contracture and maintain muscle strength in the extremities and trunk. Further, we routinely managed the gradual weaning of CSF drainage. Specifically, we considered drainage weaning approximately 2 weeks after the onset of SAH, when the acute phase of SAH, including cerebral vasospasm, was over. Further, when it was determined that CSF drainage was no longer required (e.g., when there were no symptoms of increased intracranial pressure, such as headache or unconsciousness, or enlarged ventricles, the height of the drainage disk was gradually increased, and the drainage was removed.

### Statistical analysis

We examined the relationship between sCH and the following factors: CSF drainage method (continuous or intermittent), concomitant acute hydrocephalus, age, sex, location of ruptured cerebral aneurysms, WFNS scale grade, Fisher rating scale grade, microsurgical clipping or coil embolization, cerebral vasospasm, and the use of medications (cilostazol and statin) to prevent delayed cerebral infarction due to cerebral vasospasm. Logistic regression analysis was conducted to calculate odds ratios (ORs) and 95% confidence intervals (95% CIs) for the development of sCH. The continuous variable of age at the onset of SAH was used to adjust all analyses. Further, multivariate analyses were performed after adjusting for the above-mentioned factors to assess the effect of modification and interaction between the CSF drainage method and sCH development. For continuous variables, Wilcoxon rank-sum test was used to compare mean values and standard deviations (SDs). Chi-square test was used to compare the proportions of the location of ruptured cerebral aneurysms and WFNS scale grade, etc. between the intermittent and continuous CSF drainage methods. In addition, to compare the intermittent and continuous drainage, Kaplan–Meier curves in the Cox proportional hazard regression model were plotted to demonstrate the probability of not developing sCH within 180 days after the onset of SAH. They were also evaluated after stratification based on the presence of concomitant acute hydrocephalus and concomitant intracerebral hematomas, severe initial symptoms at SAH onset, and initial pressure setting at the time of CSF drainage placement. In the present study, severe symptoms were defined by WFNS grade IV or V, whereas mild symptoms were defined by WFNS grades I–III. A high initial pressure setting was defined as 12.0 cmH_2_O or higher of the initial height of the drainage disk, whereas low pressure was defined as < 12.0 cmH_2_O. All missing variables were considered as deficit data, and no other variables were adjusted. A probability value (*P*) of < 0.05 was considered to be statistically significant. R software (version 4.2.3, R Foundation for Statistical Computing, Vienna, Austria, http://www.R-project.org) was used for all statistical analyses.

## Results

### Clinical characteristics

Table 1 summarizes the clinical characteristics of our study population. Among 204 patients with SAH (69 men and 135 women; mean age ± SD, 62.8 ± 14.7 years), 136 (49 men and 87 women; 62.8 ± 14.9 years) underwent continuous CSF drainage for an average of 10.9 ± 7.2 days, whereas 68 (20 men and 48 women; 62.8 ± 14.3 years) underwent intermittent CSF drainage for an average of 9.0 ± 5.1 days. Details of CSF drainage management are summarized in Table 2. The most common CSF drainage management was via the lumbar subarachnoid space alone, in 100 patients (49%). Continuous CSF drainage was started 0.95 ± 0.85 days after SAH onset and stopped after 11.83 ± 6.95 days, whereas intermittent CSF drainage was started 1.62 ± 1.05 after SAH onset and stopped after 10.57 ± 4.91 days. The median and range of the initial height of the disk in any CSF drainage were set at 12.0 and 0–20.0 cmH_2_O in the continuous CSF drainage group, and 10.0 and 6.0–20.0 cmH_2_O in the intermittent drainage group, with significant differences (*P* = 0.02). The two groups (continuous and intermittent CSF drainage) showed significant differences in terms of acute hydrocephalus and cerebral vasospasm prevalence, cilostazol and statin use, and WFNS scale grade for symptom severity; conversely, no significant differences were found in terms of the location of ruptured cerebral aneurysms and Fisher rating scale grades. Among 204 patients with aneurysmal SAH, 103 were treated with microsurgical clipping and 101 with coil embolization. Notably, there were no statistically significant differences in terms of these treatments between the two groups.

**Table 1:** Clinical features of the study population

	Continuous	Intermittent	*P* value
Total number	136	68	
Sex (Male: female)	49: 87	20: 48	0.433
Mean ± SD age (years)	62.8 ± 14.9	62.8 ± 14.3	0.839
<40	9	3	0.835
40–49	16	9	
50–59	30	17	
60–69	35	18	
70–79	27	9	
80<	19	12	
Aneurysm location			0.664
MCA	30	9	
ACA	43	25	
IC-PCoA	29	16	
ICA	11	6	
posterior circulation	23	12	
WFNS scale			0.004
Grade I	30	31	
Grade II	41	14	
Grade III	8	5	
Grade IV	20	10	
Grade V	37	8	
Fisher rating scale			0.452
Group 1 or 2	9	6	
Group 3	107	56	
Group 4	20	6	
Acute hydrocephalus	46	12	0.021
Cerebral vasospasm	24	4	0.029
Microsurgical clipping	71	30	0.301
Endovascular coiling	65	38	
Cilostazol usage	74	64	< 0.001
Statin usage	70	65	< 0.001

Continuous: Continuous CSF drainage

Intermittent: Intermittent CSF drainage

*P* value; Probability value of sNPH

SD; Standard deviation

**Table 2:** Cerebrospinal fluid drainage management in acute phase of aneurysmal subarachnoid hemorrhage

	With acute hydrocephalus (58)		Without acute hydrocephalus (146)		
CSF drainage via	Continuous (46)	Intermittent (12)	Continuous (90)	Intermittent (56)	Total
lumbar SAS only	17	4	47	32	100
cistern only	7	0	14	10	31
cistern followed by lumbar SAS	3	2	16	7	28
ventricles followed by lumbar SAS	8	5	5	7	25
ventricles only	8	0	6	0	14
ventricles and cistern	3	0	1	0	4
ventricles and cistern followed by lumbar SAS	0	1	1	0	2

SAS: subarachnoid space

### Efficacy of intermittent CSF drainage

Overall, 96 (47.0%) of 204 patients treated with aneurysmal SAH developed sCH at an average of 35.1 ± 19.8 (range, 15–132) days after SAH. Further, 74 (54.4%) of 136 patients managed with continuous CSF drainage developed sCH at 35.2 ± 18.6 (15–114) days after SAH, whereas 22 (32.4%) of 68 patients managed with intermittent CSF drainage developed sCH at 34.8 ± 24.0 (17–132) days after SAH.

As shown in Table 3, after multivariable adjustment, compared to continuous CSF drainage, intermittent CSF drainage was found to be associated with a significantly lower risk of sCH development (multivariate OR, 0.25; 95% CI, 0.11–0.57; *P* = 0.005). Furthermore, the presence of concomitant acute hydrocephalus (multivariate OR, 8.46; 95% CI, 3.01–23.75; *P* < 0.001) as well as the WFNS scale grades IV (multivariate OR, 5.40; 95% CI, 2.37–12.28; *P* = 0.001) and V (multivariate OR, 4.84; 95% CI, 1.78–13.14; *P* = 0.009) were associated with a significantly higher risk of sCH development. Compared with ruptured aneurysms in MCA, those in ACA, IC-PCoA, and posterior circulation were associated with a significantly higher risk of sCH development. Further, regarding the risk of sCH development in terms of treatment, there was no statistically significant difference between microsurgical clipping and coil embolization.

**Table 3:** The odds ratio of the development of sNPH after SAH

	sNPH (-)	sNPH (+)	A–OR^†^ (95% CI)	*P* value	M–OR^§^ (95% CI)	*P* value
Total number	108	96				
Female	80	55	0.35 (0.20–0.60)	0.001	0.42 (0.20–0.87)	0.049
Age (60 y.o. or older)	56	64	1.86 (1.15–2.99)	0.033	1.86 (1.15–2.99)	0.076
Intermittent CSF drainage	46	22	0.39 (0.23–0.66)	0.003	0.25 (0.11–0.57)	0.005
Endovascular coiling	58	43	0.70 (0.44–1.12)	0.210	2.45 (1.12–5.35)	0.059
Aneurysm location						
MCA	29	10	reference		reference	
ACA	29	39	5.24 (2.44–11.24)	< 0.001	9.66 (3.4–27.4)	< 0.001
IC-PCoA	24	21	2.45 (1.11–5.42)	0.063	4.18 (1.43–12.21)	0.028
ICA	10	7	2.30 (0.81–6.53)	0.188	3.6 (0.83–15.61)	0.151
posterior circulation	16	19	4.59 (1.94–10.86)	0.004	5.26 (1.39–19.92)	0.040
WFNS scale						
Grade I	46	15	reference		reference	
Grade II	38	17	1.43 (0.72–2.86)	0.392	1.32 (0.56–3.11)	0.590
Grade III	7	6	2.44 (0.85–6.96)	0.162	2.18 (0.65–7.32)	0.290
Grade IV	7	23	9.22 (3.87–21.99)	< 0.001	8.46 (3.01–23.75)	< 0.001
Grade V	10	35	10.51 (4.87–22.69)	< 0.001	4.84 (1.78–13.14)	0.009
Fisher CT rating scale						
Group 1 or 2	11	4	reference		reference	
Group 3	89	74	2.11 (0.77–5.81)	0.224	0.65 (0.20–2.12)	0.549
Group 4	8	18	6.10 (1.82–20.44)	0.014	0.84 (0.19–3.76)	0.844
Acute hydrocephalus	11	47	9.53 (5.0–18.17)	< 0.001	5.40 (2.37–12.28)	< 0.001
Cerebral vasospasm	11	17	1.66 (0.83–3.33)	0.229	1.28 (0.47–3.48)	0.681
Cilostazol usage	35	31	1.03 (0.63–1.70)	0.915	0.54 (0.23–1.24)	0.222
Statin usage	38	31	0.91 (0.55–1.49)	0.747	0.45 (0.20–1.00)	0.099

A-OR^†^; The age-adjusted odds ratio in logistic regression analysis

M-OR^§^; Multivariate odds ratio for sNPH after adjustment for age, gender, CSF drainage method, treatment method, aneurysm location, WFNS scale, Fisher CT rating scale, acute hydrocephalus, cerebral vasospasm, cilostazol usage, and statin usage

**Fig. 3:**
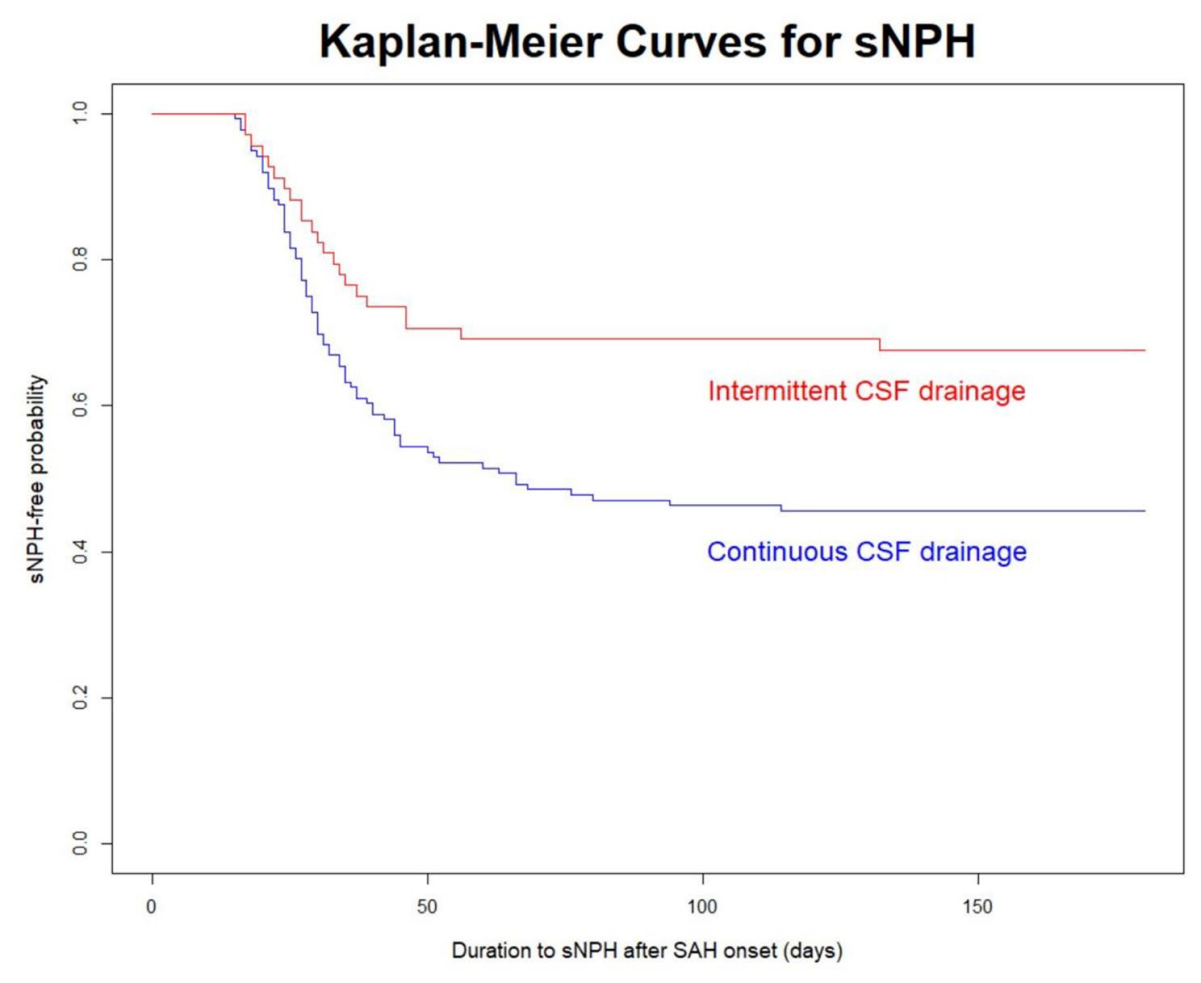
Kaplan–Meier curves for the different CSF drainage methods

The Kaplan–Meier curve for the probability of not developing sCH within 180 days after the onset of SAH are presented in Fig. 3. Briefly, intermittent CSF drainage was less likely to cause sCH than continuous CSF drainage.

**Fig. 4:**
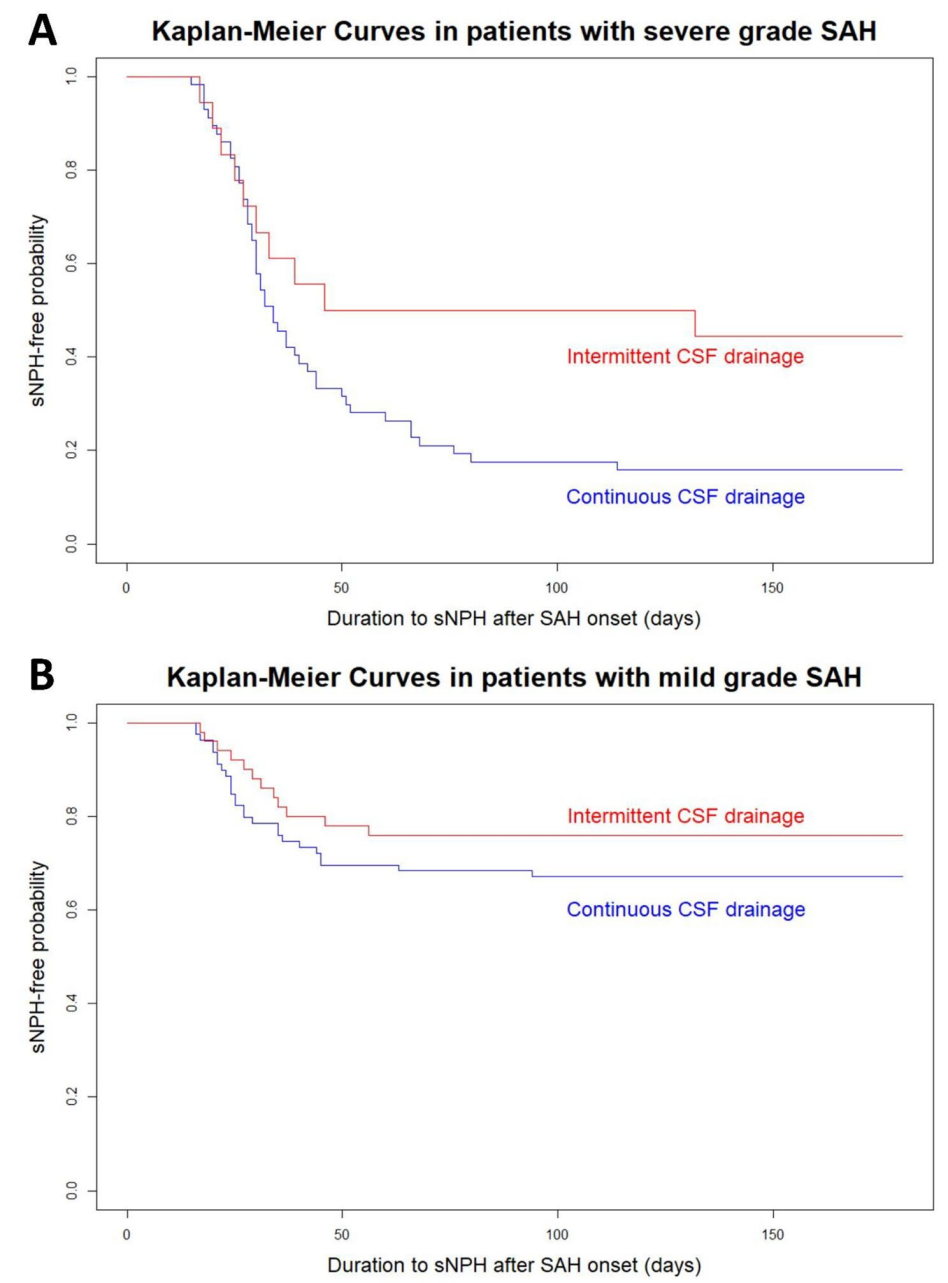
Kaplan–Meier curves for the different CSF drainage methods in the subgroups of severe-grade SAH (**A**) and mild-grade SAH (**B**)

**Fig. 5:**
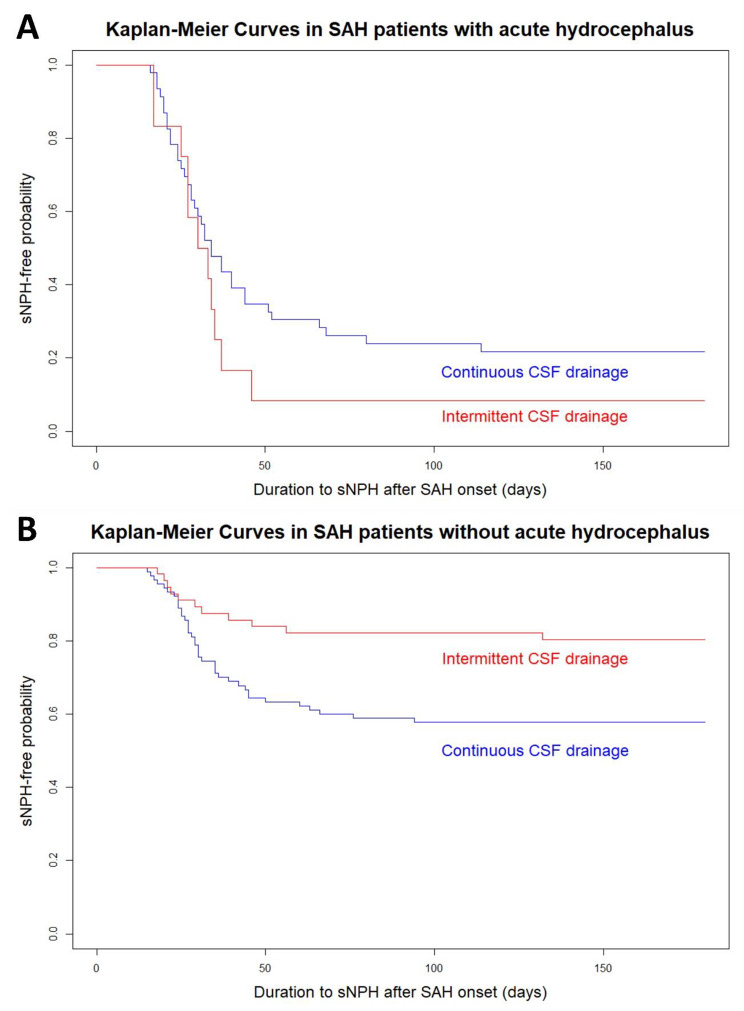
Kaplan–Meier curves for the different CSF drainage methods in the subgroups of SAH with (**A**) and without (**B**) acute hydrocephalus

**Fig. 6:**
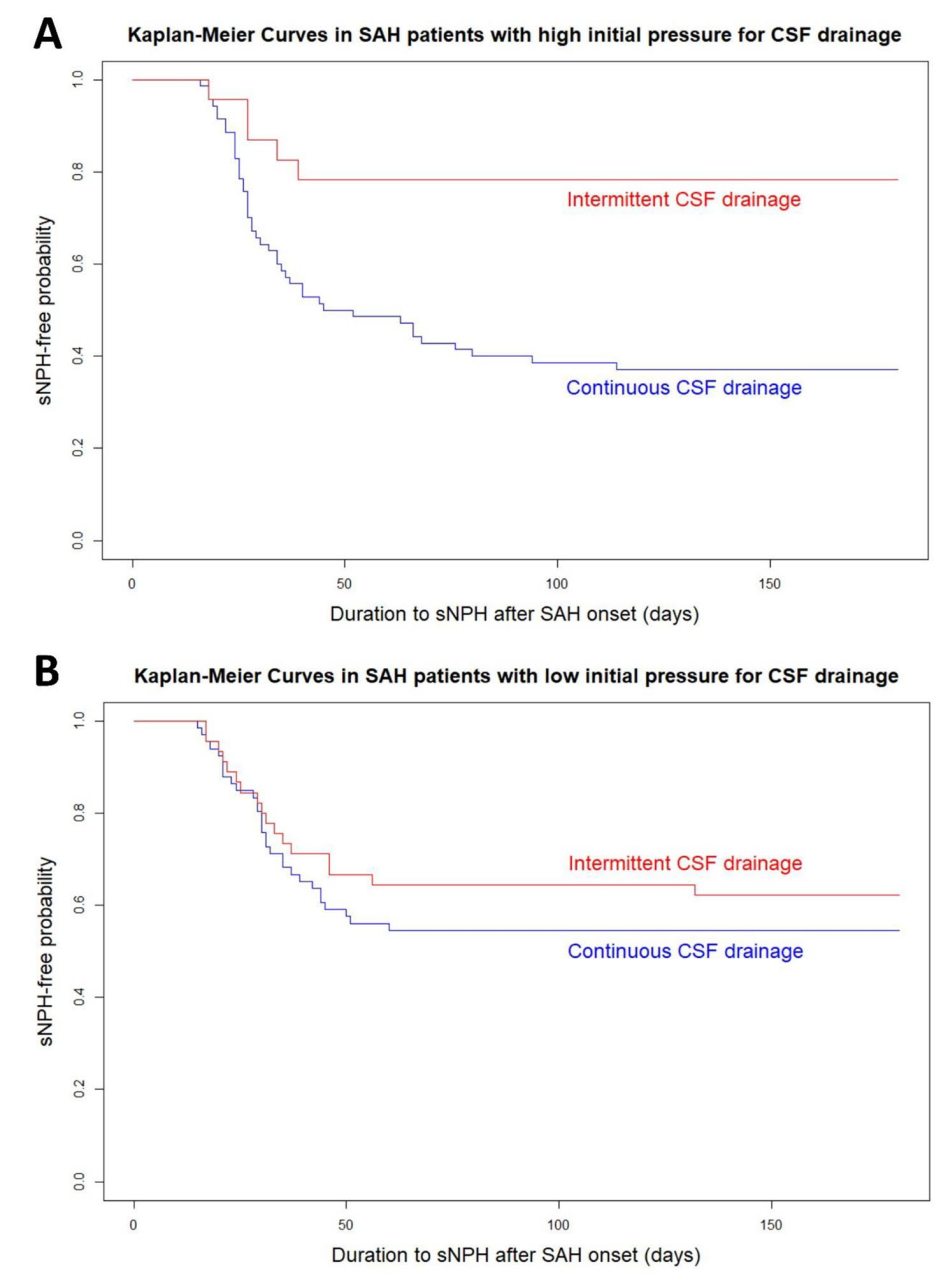
Kaplan–Meier curves for the different CSF drainage methods in the subgroups of high (**A**) and low (**B**) initial pressure setting for CSF drainage

**Fig. 7:**
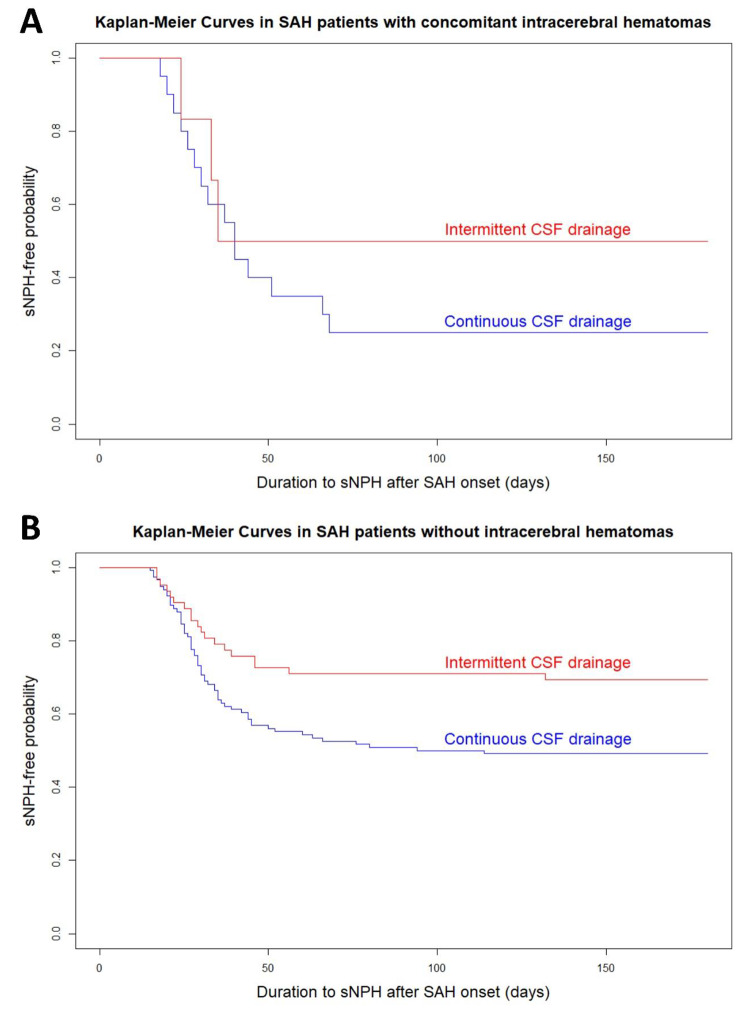
Kaplan–Meier curves for the different CSF drainage methods in the subgroups of SAH with and without intracerebral hematomas

Moreover, in the subgroup analyses, the intermittent drainage had a lower tendency to cause sCH in both subgroups—severe-grade SAH (grade IV or V on the WFNS scale) and mild-grade SAH (grades I–III on the WFNS scale)—although the probability of developing sCH was higher and the effect of intermittent CSF drainage was greater in the severe-grade SAH subgroup than in the mild-grade SAH subgroup (Fig. 4). The age- and multivariate-adjusted ORs of intermittent CSF drainage in the severe-grade SAH group were 0.23 (95% CI, 0.08–0.62; *P* = 0.015) and 0.20 (95% CI, 0.04–0.95; *P* = 0.088), and those in the mild-grade SAH were 0.66 (95% CI, 0.33–1.29; *P* = 0.307) and 0.33 (95% CI, 0.12–0.95; *P* = 0.088), respectively.

In the acute hydrocephalus subgroup (Fig. 5), the probability of developing sCH was nearly the same until approximately 30 days after the onset of SAH, regardless of whether intermittent or continuous CSF drainage was performed. However, after 30 days, the probability was significantly higher in patients managed with intermittent CSF drainage. Conversely, in intermittent CSF drainage, the probability of developing sCH was lower in the subgroup without acute hydrocephalus (Fig. 5). The age- and multivariate-adjusted ORs of intermittent CSF drainage in the subgroup with acute hydrocephalus were 3.10 (95% CI, 0.50–19.3; *P* = 0.308) and 8.37 (95% CI, 0.56–125.2; *P* = 0.196), and those in the subgroup without acute hydrocephalus were 0.32 (95% CI, 0.16–0.63; *P* = 0.005) and 0.11 (95% CI, 0.04–0.31; *P* < 0.001).

In the subgroup of high initial pressure of CSF drainage (Fig. 6), intermittent CSF drainage had a significantly lower probability of developing sCH, compared with continuous drainage. On the other hand, the subgroup of low initial pressure was the same probability of developing sCH for both intermittent and continuous CSF drainage up to 30 days after SAH onset, but after 30 days, intermittent CSF drainage was less likely to develop sCH. The age- and multivariate-adjusted ORs of intermittent CSF drainage in the subgroup of high initial pressure of CSF drainage were 0.15 (95% CI, 0.06–0.38; *P* = 0.001) and 0.02 (95% CI, 0.01–0.11; *P* < 0.001), and those in the subgroup of low initial pressure of CSF drainage were not significant.

In the subgroup of concomitant intracerebral hematomas, i.e., grade 4 on Fisher CT rating scale at admission (Fig. 7), the probability of developing sCH was nearly the same until approximately 30 days after the onset of SAH, regardless of intermittent or continuous CSF drainage was performed. However, after 30 days, intermittent CSF drainage was less likely to develop sCH. On the other hand, in the subgroup of SAH without intracerebral hematomas, intermittent CSF drainage had a significantly lower probability of developing sCH, compared with continuous drainage. The age- and multivariate-adjusted ORs of intermittent CSF drainage in the subgroup of SAH without intracerebral hematoma were 0.40 (95% CI, 0.23–0.70; *P* = 0.007) and 0.22 (95% CI, 0.10–0.49; *P* < 0.001), although those in the subgroup of concomitant intracerebral hematomas were not significant.

## Discussion

The present study found that intermittent CSF drainage is more effective than continuous CSF drainage in reducing the development of sCH after aneurysmal SAH. To the best of our knowledge, only one previous study has demonstrated the effectiveness of intermittent CSF drainage. In the study by Rao et al. [18], intermittent CSF drainage with rapid weaning of external ventricular drainage resulted in a fewer cases of sCH requiring permanent placement of the VPS compared to continuous CSF drainage with gradual weaning. Further, the same study group recently conducted a randomized multicenter controlled trial to compare rapid and gradual weaning of external ventricular drainage at six neurocritical care units in the United States [19]. Their study provided positive evidence that the rapid weaning protocol was associated with a lower rate of VPS placement, but they did not investigate the effectiveness of intermittent CSF drainage. However, the efficacy of rapid weaning of external ventricular drainage remains controversial [19]. For example, an observational cohort study with pooled data from two German university hospitals provided conflicting evidence that compared to rapid weaning, gradual weaning of external ventricular drainage reduces the risk of sCH after SAH [20]. In the present study, we could demonstrate the effectiveness of intermittent drainage alone in reducing sCH development because our study protocol included intermittent CSF drainage with gradual weaning of external CSF drainage. In addition, we reported that intermittent CSF drainage was associated with reduction in sCH development, regardless of the severity of symptoms at the time of SAH onset, initial pressure setting at the time of emergency CSF drainage placement and concomitant intracerebral hematomas. Further, in patients with SAH and concomitant acute hydrocephalus, compared to continuous CSF drainage, intermittent CSF drainage was not effective in preventing sCH, but rather increased the long-term risk of developing sCH. Therefore, we conclude that intermittent CSF drainage is not recommended in acute hydrocephalus-concomitant SAH.

Although the mechanism of the effectiveness of intermittent CSF drainage in reducing sCH development is unknown, storage of CSF in the subarachnoid space during periods of no drainage would help prevent arachnoid membrane adhesion. There is consensus that extensive adhesions in the subarachnoid space are the main pathogenesis of sCH after SAH [11, 21–24]. Further, because of the effectiveness of lumbar drainage for complications of subcutaneous effusions after craniotomy, keeping the subarachnoid space as dry as possible seems reasonable to promote subarachnoid space adhesion. The total volume of daily CSF drainage was set at approximately 150 mL for both continuous and intermittent drainage, possibly supporting the hypothesis that temporary CSF accumulation in the subarachnoid space inhibits subarachnoid space adhesion. Furthermore, it is unclear why intermittent CSF drainage did not reduce the risk of developing sCH in cases of acute hydrocephalus in combination with SAH. Meanwhile, the pathogenesis of acute hydrocephalus may be related to the pathogenesis of chronic hydrocephalus; thus, it is possible for acute hydrocephalus to prolong and become chronic sCH. Another hypothesis states that acute hydrocephalus causes a pressure imbalance between the ventricles and subarachnoid space, compressing the brain from inside to outside, and that the pressure imbalance becomes chronic, leading to the development of sCH.

Furthermore, this study confirmed that increasing age, severe initial symptoms, a large amount of diffuse subarachnoid hematoma, the presence of concomitant acute hydrocephalus, and the location of ruptured cerebral aneurysms in ACA, IC-PCoA, and posterior circulation were significantly associated with the development of sCH, and these results are consistent with those of previous studies [1–14]. However, in this study, there were no significant differences in treatment methods and the prevalence of sCH development after SAH. A previous study reported that coil embolization was more effective than microsurgical clipping alone in reducing the risk of sCH [11]. In addition, there was a significant difference in cilostazol and statin use between the intermittent and continuous CSF drainage groups in this study. This discrepancy may be attributed to the time overlap between the initiation of cilostazol and statin use to prevent delayed cerebral infarction due to cerebral vasospasm and the initiation of intermittent CSF drainage.

This study has several limitations. First, there are different historical backgrounds on the treatment of SAH, including CSF drainage, as mentioned above. Advances in endovascular therapy and frequency of use of cilostazol and statins markedly differ between before and after 2012, and their influences should not be overlooked. Second, 74 patients (26.6%) of 278 consecutive patients with SAH were excluded from the present study due to various reasons. Third, there was a difference in the number of cases between the two groups, with 136 patients managed with continuous CSF drainage and 68 patients managed with intermittent CSF drainage. This difference may be attributed to selection bias. Finally, because this study was conducted at a single institute in a retrospective fashion, we could not establish a direct causal relationship between intermittent CSF drainage and the development of sCH. Thus, further randomized multicenter controlled trials are warranted to assess the effectiveness of new interventions or treatments.

## Conclusions

The results of the present study indicate that intermittent CSF drainage after SAH is associated with fewer cases of sCH than continuous CSF drainage. Although intermittent CSF drainage was ineffective in SAH with concomitant acute hydrocephalus, it was effective in reducing sCH development regardless of the severity of the initial symptoms at the onset of SAH. Further studies are required to understand the mechanisms of the effectiveness of intermittent CSF drainage in reducing sCH development after SAH.

## Data Availability

The data including clinical information will only be available if the ethics committees approve new participation in the collaborative research.
